# Interobserver variation in classifying lymphomas among hematopathologists

**DOI:** 10.1186/s13000-014-0162-3

**Published:** 2014-08-22

**Authors:** Tawatchai Pongpruttipan, Sanya Sukpanichnant, Thamathorn Assanasen, Lertlakana Bhoopat, Kanita Kayasut, Wasana Kanoksil, Pongsak Wannakrairot

**Affiliations:** Department of Pathology, Faculty of Medicine Siriraj Hospital, Mahidol University, 10330 Bangkok, Thailand; Department of Pathology, Faculty of Medicine, Chulalongkorn University, Bangkok, Thailand; Department of Pathology, Faculty of Medicine, Chiang Mai University, Bangkok, Thailand; Department of Pathology, Faculty of Medicine, Prince of Songkla University, Bangkok, Thailand; Department of Pathology, Faculty of Medicine Ramathibodi Hospital, Mahidol University, Bangkok, Thailand

**Keywords:** Discordance, Disagreement, Interobserver variation, Pitfall, Hematopathologist, Lymphoma, WHO classification

## Abstract

**Background:**

Lymphomas are common malignancies that have various subtypes with many overlapping histologic, immunophenotypic and genetic features. Therefore, discordance in classifying lymphoma among pathologists may be encountered. But this issue is not well characterized. We conducted the present study to demonstrate discordances among Thai hematopathologists as well as to highlight common arguing points for classifying lymphomas.

**Methods:**

The 117 lymphoma cases were randomly retrieved and individually reviewed by 7 hematopathologists, members of the “Thai Hematopathologist Group,” without knowing the original diagnoses. The consensus diagnoses were given from a discussion by all members. In each case, the diagnosis from each participant was compared with the consensus diagnosis and classified into 4 categories as follow: 1) concordance, 2) minor discordance, 3) major discordance and 4) serious discordance.

**Results:**

There were approximately 11% discordances between original and consensus diagnoses. The average discordances among all pathologists according to minor, major and serious discordances were 10%, 3.5% and 0.3%, respectively. Diffuse large B-cell lymphoma had the least discordance (7%). Small biopsies had been found to increase discordances in some lymphoma subtypes.

**Conclusions:**

The present study reveals some degrees of interobserver variation in classifying of lymphoma by using the 2008 WHO classification among hematopathologists. Some types of lymphomas on small biopsies were found to have a significant higher discordance rate. This study also described some common diagnostic discordances regarded as potential pitfalls in classifying lymphomas.

**Virtual Slides:**

The virtual slide(s) for this article can be found here: http://www.diagnosticpathology.diagnomx.eu/vs/13000_2014_162

## Background

Lymphomas are common malignancies worldwide. Due to the advances in immunology and genetic technologies, classification of lymphoma has been regularly updated [1,2]. In the current WHO classification published in 2008, clinical features, histopathology, immunophenotype and genetic features are important to classify lymphomas [1,2]. Since, there were >30 lymphoma subtypes in the 2001 version and >60 in the 2008 version. Such many entities have increased the overlapping clinicopathologic features thus may increase difficulty in classifying lymphomas. As previously reported, approximately 10% of T-cell lymphomas were either misdiagnosed or inadequately subclassified by world expert hematopathologists [3].

Interobserver variation as well as pitfalls in classifying all lymphoma subtypes according to the WHO classification among hematopathologists has not been well documented. We hereby conducted this study to address this issue by allowing 7 Thai hematopathologists, members of the “Thai Hematopathologist Group”, to individually review 117 lymphoma cases in order to determine the variation in classifying lymphomas. The results of the present study may be useful for hematologists and hematopathologists alike to aware common diagnostic pitfalls.

## Methods

This study was approved by Institutional Review Board of each institution participating in the study, including Faculty of Medicine Siriraj Hospital, Mahidol University, Faculty of Medicine, Chulalongkorn University, Faculty of Medicine, Chiang Mai University, and Faculty of Medicine, Prince of Songkhla University.

The studied cases were recruited from cases with original diagnoses of Hodgkin and non-Hodgkin lymphomas. Mycosis fungoides was excluded from the study. A hundred and nineteen cases were randomly recruited from 4 institutions, but 2 cases were excluded due to inadequate material, thus 117 remained (58 from Chulalongkorn Hospital, 48 from Siriraj Hospital, 8 from Songkhla University Hospital, and another 8 Chiang Mai University Hospital). The cases were selected consecutively, regardless of lymphoma subtypes. Each case was originally diagnosed by either general pathologists or hematopathologists of each institute. All the original diagnoses were made mainly based on evaluation of Hematoxylin and Eosin (H&E) in conjunction with immunohistochemistry (IHC) slides. Some cases also had additional studies, such as EBV-encoded small RNA (EBER) in situ hybridization, kappa and lambda immunoglobulin light chain in situ hybridization, T-cell receptor gene and/or immunoglobulin heavy chain gene rearrangement analysis.

There were 7 hematopathologists, members of the “Thai Hematopathologist Group”, participating to review the cases. We set up two days for meeting. On the first day, there was a 6-hour period for each participant to individually review all 117 cases that provided only H&E, IHC and in situ hybridization slides. All of clinical data, original diagnoses and pathological reports were not given. Skipping cases without giving diagnosis was not permitted. On the second day of meeting, discussion and consensus on cases with discordance were conducted by all hematopathologists, using microscope with real-time video projector. Each diagnosis given by participants was categorized into 4 categories toward its consensus diagnosis (Table 1). Difficult cases were defined as cases with ≥40% of discordance among participating hematopathologists. Of the 117 cases, 4 were initially unable to make the consensus. After the meeting, additional immunohistochemical staining with or without EBER in situ hybridization were performed and the consensus diagnoses were reached.

**Table 1:** **Definition of each diagnostic category made by hematopathologists when compared to the consensus diagnosis**

**Category**	**Description**
1. Concordance	Hematopathologist made a single diagnosis, identical to the consensus diagnosis
2. Minor discordance	Hematopathologist made >1 diagnoses, and one of the given differential diagnoses met the consensus diagnosis; or discordance between classical Hodgkin lymphoma subtypes
3. Major discordance	Hematopathologist made ≥1 diagnosis(es) of lymphoma, but not any of given diagnosis met the consensus diagnosis
4. Serious discordance	Hematopathologist made a diagnosis of reactive process, disagreed to the consensus diagnosis of lymphoma

Kappa statistics of the five most common lymphoma subtypes of each pathologist was also analyzed.

## Results

Within a 6-hours period given, hematopathologists had variably speeds in reviewing slides, ranged from 56–117 cases (Table 2). All consensus diagnoses were lymphomas. In detail, for the 4 cases that consensus diagnoses were not reached initially, 2 were diffuse large B-cell lymphoma (DLBCL) which required additional IHC for distinguishing from Burkitt lymphoma (BL), while other 2 were extranodal NK/T-cell lymphoma, nasal type (ENKTL) which required additional IHC and EBER in situ hybridization for diagnostic confirmation.

**Table 2:** **Number of reviewed cases and percentage in each diagnostic category of each hematopathologist**

	**Hematopathologist** ^**a**^							**Average**
	**A**	**B**	**C**	**D**	**E**	**F**	**G**	
**Numbers of reviewed cases** ^**b**^	117	105	56	94	88	114	81	94 (80%)
**Category of diagnoses** ^**c**^ **(%)**								
1. Concordance	84	84	86	92	90	84	85	86
2. Minor discordance	13	12	9	6	7	14	6	10
3. Major discordance	3	4	5	2	3	1	7	3.5
4. Serious discordance	0	0	0	0	0	1	1	0.3

^a^Seven hematopathologists were blindly coded as A to G.

^b^Numbers of reviewed cases are numbers of cases that each hematopathologist could individually review within a 6-hours period.

^c^Please see the description of each diagnostic category in Table 1.

When compared to the consensus diagnoses, each participant had a similar percentage of concordances, ranging from 84-92% with 86% in average (Table 2). The average frequencies of minor, major, and serious discordances were 10%, 3.5% and 0.3%, respectively (Table 2). Frequencies of each lymphoma subtype and their frequencies of discordances were shown in Table 3.

**Table 3:** **Summary of discordance for each lymphoma subtype made by all hematopathologists**

**Consensus diagnosis**	**Overall frequency (%)**	**Category of diagnoses** ^**a**^ **(%)**			
		**Concordance**	**Minor discordance**	**Major discordance**	**Serious discordance**
Diffuse large B-cell lymphoma	68 (58)	93	4	3	0
Follicular lymphoma	7 (6)	77	20	3	0
MALT lymphoma	6 (5)	79	12	9	0
Nodular sclerosis classical HL	6 (5)	81	19	0	0
Extranodal NK/T-cell lymphoma	5 (4)	65	23	12	0
Nodal marginal zone lymphoma	3 (3)	72	16	6	6
Mixed cellularity classical HL	3 (3)	59	41	0	0
Mantle cell lymphoma	3 (3)	100	0	0	0
Peripheral T-cell lymphoma, NOS	3 (3)	75	13	6	6
Angioimmunoblastic T-cell lymphoma	2 (2)	90	10	0	0
Anaplastic large cell lymphoma	2 (2)	91	0	9	0
T lymphoblastic lymphoma	2 (2)	78	22	0	0
Lymphoplasmacytic lymphoma	2 (2)	18	55	27	0
Classical HL	1 (1)	100	0	0	0
Lymphocyte-rich classical HL	1 (1)	33	67	0	0
Marginal zone/lymphoplasmacytic lymphoma^b^	1 (1)	100	0	0	0
Subcutaneous panniculitis-like T-cell lymphoma	1 (1)	100	0	0	0
Cutaneous T-cell lymphoma, unclassified	1 (1)	75	25	0	0
Average		86	10	3.5	0.3

^a^Please see the description of each diagnostic category in Table 1.

^b^A CD5-/CD10-/cyclin D1- small B-cell neoplasm with plasmacytic differentiation in a bone marrow biopsy which definite subtype could not be classified.

Five most common lymphomas in the present study were DLBCL, follicular lymphoma (FL), extranodal marginal zone lymphoma of mucosa-associated lymphoid tissue (MALT lymphoma), nodular sclerosis classical Hodgkin lymphoma (NSCHL), and ENKTL. For DLBCL, about half of the discordances were BL while one-third was FL. For MALT lymphoma, 7/8 of discordances were various small B-cell lymphoma subtypes, while the other was “DLBCL versus FL”. For FL, 5/9 of discordances were associated with other small B-cell lymphoma subtypes, particularly with nodal marginal zone B-cell lymphoma (NMZL), while 4/9 were debated with DLBCL or with accompanying DLBCL. In addition, 3 out of 7 FLs were small incisional biopsies which had significantly higher discordances when compared to the excisional cases (7/9 vs. 2/19, p = 0.02). For NSCHL, all discordances were debated on its various subtypes.

The difficult cases, defined by using the aforementioned criteria, accounted for 16% (19/117) of all cases were demonstrated in details in Table 4. Of interest, these difficult cases accounted for 71% of all discordances made.

**Table 4:** **Details of the difficult cases** ^**a**^

**Case no.**	**Consensus diagnosis**	**Discordance of the original diagnosis** ^**b**^	**Frequency of each diagnostic category** ^**c**^			
			**Concordance**	**Minor discordance**	**Major discordance**	**Serious discordance**
86	MALT lymphoma	Minor	0	2	3	0
101	Extranodal NK/T-cell lymphoma^d^	Major	0	1	3	0
19	Diffuse large B-cell lymphoma^d^	-	4	0	3	0
83	Lymphoplasmacytic lymphoma	Minor	0	2	2	0
32	Diffuse large B-cell lymphoma	-	3	0	2	0
82	Lymphoplasmacytic lymphoma	Minor	2	4	1	0
113	Peripheral T-cell lymphoma, NOS	Major	1	2	1	0
78	Follicular lymphoma	-	3	2	1	0
36	Diffuse large B-cell lymphoma	-	0	5	0	0
100	Extranodal NK/T-cell lymphoma^d^	Minor	0	5	0	0
93	Mixed cellularity classical HL	Minor	1	5	0	0
76	Follicular lymphoma	-	3	4	0	0
109	Nodular sclerosis classical HL	Major	3	4	0	0
98	Nodal marginal zone lymphoma	-	3	3	0	0
84	Lymphocyte-rich classical HL	Minor	1	2	0	0
91	Mixed cellularity classical HL	Minor	3	2	0	0
62	Diffuse large B-cell lymphoma^d^	-	3	2	0	0
116	T lymphoblastic lymphoma	-	3	2	0	0
23	Diffuse large B-cell lymphoma	-	1	1	0	0
Total			23	30	11	0

^a^Difficult cases were cases that at least 40% of hematopathologists (participants) made discordant diagnoses.

^b^Original diagnoses, initially made by attending pathologists in clinical services, were also categorized into 4 categories by comparing to the consensus diagnoses. Blanks represent “concordance”.

^c^Please see the description of each diagnostic category in Table 1.

^d^Cases that consensus diagnoses could not be reached at the initial meeting and additional investigations were performed before reaching the consensus diagnoses.

Compared to the consensus diagnoses, 6% (7/117) of original diagnoses were with minor discordance and 5% (6/117) with major discordance (Table 3). No original diagnosis with serious discordance was found. Among the 13 cases with discordant original diagnoses, 11 of them (85%) also had high discordant rate, fulfilling the criteria of “difficult cases”.

In the present study, cases with “serious discordance” were rare. One out of 2 was a NMZL that was misdiagnosed as lymph node with marginal zone hyperplasia by one participant. Another was a case of peripheral T-cell lymphoma, not otherwise specified (PTCL, NOS) that was misdiagnosed as atypical lymphoid hyperplasia.

The kappa statistics of the five most common lymphoma subtypes for each pathologist were shown in Table 5. The overall kappa statistics for DLBCL, FL, MALT lymphoma, NSCHL and ENKTL were 0.90, 0.80, 0.84, 0.75 and 0.74, respectively.

**Table 5:** **Kappa statistics of the five most common lymphoma subtypes of each hematopathologist**

	**Kappa statistics of each hematopathologist (n)**							**Average**
	**A**	**B**	**C**	**D**	**E**	**F**	**G**	
Diffuse large B-cell lymphoma	0.93 (68)	0.88 (60)	0.85 (38)	0.96 (57)	0.95 (47)	0.93 (66)	0.92 (50)	0.92
Follicular lymphoma	0.82 (7)	0.75 (6)	0.45 (3)	0.88 (5)	0.92 (7)	0.82 (7)	0.79 (5)	0.80
MALT lymphoma	0.90 (6)	0.65 (6)	0.79 (3)	0.90 (6)	1.00 (4)	0.90 (6)	1.00 (2)	0.84
Nodular sclerosis classical HL	0.90 (6)	0.90 (6)	0 (1)	0.90 (6)	0.92 (6)	0.65 (6)	1.00 (2)	0.75
Extranodal NK/T-cell lymphoma	0.74 (5)	0.85 (4)	1.00 (3)	1.00 (3)	0.49 (3)	0.74 (5)	0.49 (1)	0.74

## Discussion

Similar to the present study, other studies also reported a common interobserver variation in classifying lymphomas using other classifications or by various ways [4–7]. The overlapping features among lymphoma subtypes together with either interobserver or inter-institutional variation inevitably lead to discordance [8–10]. In the current WHO classification (2008), many subtypes of lymphomas use mostly the same criteria as those described in the 2001 version, while some underwent critical changes (such as grade 3 FL). Certainly, some new entities were introduced to the current 2008 version. Generally, effort to classify into more subtypes with detail in criteria should reduce discordant diagnoses. Nevertheless, in practical, discordance in diagnosis still exists and may be due to the frequent overlapping features in many subtypes. A recent study showed an evidence of decreased discordant rate. The authors hypothesized that this may be due to more experience of the pathologists in applying the WHO classification [11].

Distribution of each lymphoma subtype in the present study was similar to the previous study of Thai lymphomas [12]. This suggests a relatively well random selection of cases. There were some limitations on the present study as follow: 1) Only a 6-hours period might remarkably limits participants to diagnose 117 cases very carefully, although they were not forced to review all persisting cases; 2) Lacking of giving clinical information might diminish accurate diagnosis in some cases; 3) All participants knew that all recruited cases were originally diagnosed lymphoma, hence less chance for making diagnosis of reactive process; and 4) No results of ancillary studies were provided to the participants.

The present study gave all available slides to the participants, so it is somewhat similar to a process in reviewing pathological diagnosis, except for lacking of provided original diagnosis, clinical information and other ancillary studies. However, the aim of the present study is mainly concentrated to the interobserver variation among hematopathologists. As shown in the results, discordances between the original diagnosis and consensus diagnosis were slightly less than the average of those made by participants, which may be due to the aforementioned limitations.

From each participant, overall discordance of any categories ranged from 8-16%. Almost all were minor and major discordances. The summation of these 2 categories was relatively similar among participants. However, it is not obviously related to the participants’ reviewing speeds. These may reflect the personal confidence in making diagnoses, whether some participants preferred to make the most likely single diagnosis or some preferred to include multiple differential diagnoses. A recent study that used WHO classification showed 9% discordant rate, lower than the present study. This may be caused by several factors including the aforementioned limitation as well as the methodological differences, for example, the former study grouped all T- and NK-cell lymphomas into a single category [11].

In the present study, only viewpoints upon discordances among the five most lymphoma subtypes was discussed. Among the five most common lymphomas, DLBCL had the highest concordance. Similar results among general pathologists have been documented [7,13]. In the present study, cases with discordance between DLBCL and BL may represent the unclassifiable B-cell lymphoma with intermediate features between DLBCL and BL in the 2008 WHO classification (2008) [14]. Grade 3 FL with any obvious diffuse growth pattern should be classified as DLBCL in the WHO classification [15]; however, some cases in the present study might have vague diffuse areas leading to diagnostic discordance. Fortunately, treatment for grade 3 FL and DLBCL is similar. In grade 3b FL, the neoplastic cells may express IRF4/MUM1 but not CD10 [16]. Other than CD10 and BCL6, HGAL might be a new sensitive marker for germinal center phenotype [17]. Furthermore, in small biopsies, it is a limitation for complete evaluation of nodular architecture, especially in cases with very large irregular neoplastic nodules. IHC markers will be helpful to highlight large neoplastic cells growing in follicular dendritic meshworks in order to reach the diagnosis of this particular FL.

A relatively high rate of discordance in making diagnosis of MALT lymphoma has been reported [6,7]. In the present study, the discordance was raised because participants mainly included other small B-cell lymphomas for differential diagnoses. Although almost cases generally lack of CD5 expression, a small subset of MALT lymphoma may express CD5 [18,19]. This may potentially increase discordance rate. Interestingly, one common pitfall is a misdiagnosis of MALT lymphoma with increased scattered large cells as DLBCL [7], that will be concerned as a significant therapeutic alteration. Only cases with definite confluent sheet of large B-cells should be diagnosed as DLBCL, either de novo or histologic transformed case [20]. And it has been documented that high proliferative index by using Ki67 is useful for distinguishing gastric DLBCL from gastric MALT lymphoma [21].

In the present study, FL cases with small incisional biopsies showed significantly higher discordances than those with excisional biopsies. Therefore, making diagnosis on cases with small incisional biopsy should be more careful. For the low-grade lymphoma cases, the discordances were mostly associated with other small B-cell lymphoma subtypes; while the high-grade FL was mostly debated between DLBCL as mentioned above.

For NSCHL, particularly those with small biopsies, distinctions between its subtypes were difficult and subjective. Although there are common discordances among subtyping of classical Hodgkin lymphoma, management among the subtypes is the same.

Although EBV positivity is characteristic and specific for ENKTL than other T-cell lymphoma subtypes [22,23], the discordance for ENKTL was relatively high in the present study because 2/5 cases (originally reported as PTCL, NOS and PTCL, NOS versus ENKTL) had no prior EBV studies; so participants included at least 2 differential diagnoses. EBER in situ hybridization was subsequently performed following the consensus, and both cases were positive. Study for EBV is essential for ENKTL, since ENKTL is common NK- and T-cell lymphomas in many Asian countries including Thailand [24,25]. Furthermore, ENKTL may be of either αβ or γδ T-cell lineage [26], and requires different chemotherapy regimens [27–29], as well as benefits from radiation plus therapy [26,27]. Thus, evidence of EBV association by using EBER in situ hybridization is recommended for all CD3-positive mature lymphomas. EBV LMP1 and LMP2 immunohistochemistry is positive in some proportion of cases [30], but is not a sensitive test.

Lymphoplasmacytic lymphoma, when without available clinical correlation, was one of lymphomas with a very high discordance rate as shown in this study. This may be due to the lack of specific morphologic and immunophenotypic features but with many overlapping features with other small B-cell neoplasms with plasmacytic differentiation, particularly marginal zone lymphomas with plasmacytic differentiation. Characteristic cytogenetic findings of marginal zone lymphoma, if present, might be a useful feature for dissolving of this issue.

## Conclusion

There are some degrees of interobserver variation in classifying of lymphoma by using the 2008 WHO classification. The uncommon discordances showed in the present study are similar to the previously published studies. The overlapping in morphologic, immunophenotypic and genetic features among various lymphoma subtypes may cause uncommon discordances in classifying lymphomas. Awareness of the overlapping features as well as the common pitfalls might be helpful for reducing discordances among pathologists and hematologists. Some lymphoma subtypes on small biopsies were found to have a significant higher discordance rate, particularly for those which needs architectural evaluation.

## Consent

Written informed consent was obtained from the patient for the publication of this report and any accompanying images.

## Abbreviations

BL: Burkitt lymphoma
DLBCL: Diffuse large B-cell lymphoma
EBER: EBV-encoded RNA
FL: Follicular lymphoma
H&E: Hematoxylin and Eosin
IHC: Immunohistochemistry
MALT lymphoma: Extranodal marginal zone lymphoma of mucosa-associated lymphoid tissue
ENKTL: Extranodal NK/T-cell lymphoma, nasal type
NMZL: Nodal marginal zone B-cell lymphoma
NSCHL: Nodular sclerosis classical Hodgkin lymphoma
PTCL: NOS, Peripheral T-cell lymphoma, not otherwise specified
